# Health Alliance for prudent antibiotic prescribing in patients with respiratory tract infections (HAPPY AUDIT) -impact of a non-randomised multifaceted intervention programme

**DOI:** 10.1186/1471-2296-12-52

**Published:** 2011-06-20

**Authors:** Lars Bjerrum, Anders Munck, Bente Gahrn-Hansen, Malene Plejdrup Hansen, Dorte Ejg Jarbol, Gloria Cordoba, Carl Llor, Josep Maria Cots, Silvia Hernández, Beatriz González López-Valcárcel, Antonia Pérez, Lidia Caballero, Walter von der Heyde, Ruta Radzeviciene, Arnoldas Jurgutis, Anatoliy Reutskiy, Elena Egorova, Eva Lena Strandberg, Ingvar Ovhed, Sigvard Mölstad, Robert Vander Stichele, Ria Benko, Vera Vlahovic-Palcevski, Christos Lionis, Marit Rønning

**Affiliations:** 1 Research Unit for General Practice and Section of General Practice, Department of Public Health, University of Copenhagen, Copenhagen, Denmark; 2 Research Unit of General Practice, Institute of Public Health, University of Southern Denmark, Odense, Denmark; 3 Spanish Society of Family Medicine, Barcelona, Spain; 4 University of Las Palmas, Gran Canarias, Las Palmas, Spain; 5 Misiones Association of General Family Medicine, Posadas, Argentina; 6 Public Health Department, Faculty of Health Sciences, Klaipeda University, Lithuania; 7 Association of Family Doctors, Kaliningrad, Russia; 8 Department of Clinical Sciences, Lund University, Malmoe, Sweden; 9 European Drug Utilisation Research Group, Ghent, Belgium; 10 World Organisation of Family Doctors (WONCA) Europe, Ljubljana, Slovenia; 11 World Health Organisation, Collaborating Centre for Drug Statistics Methodology, Oslo, Norway

## Abstract

**Background:**

Excessive use of antibiotics is worldwide the most important reason for development of antimicrobial resistance. As antibiotic resistance may spread across borders, high prevalence countries may serve as a source of bacterial resistance for countries with a low prevalence. Therefore, bacterial resistance is an important issue with a potential serious impact on all countries. Initiatives have been taken to improve the quality of antibiotic prescribing in primary care, but only few studies have been designed to determine the effectiveness of multifaceted strategies across countries with different practice setting. The aim of this study was to evaluate the impact of a multifaceted intervention targeting general practitioners (GPs) and patients in six countries with different health organization and different prevalence of antibiotic resistance.

**Methods:**

GPs from two Nordic countries, two Baltic Countries and two Hispano-American countries registered patients with respiratory tract infections (RTIs) in 2008 and 2009. After first registration they received individual prescriber feedback and they were offered an intervention programme that included training courses, clinical guidelines, posters for waiting rooms, patient brochures and access to point of care tests (Strep A and C-Reactive Protein). Antibiotic prescribing rates were compared before and after the intervention.

**Results:**

A total of 440 GPs registered 47011 consultations; 24436 before the intervention (2008) and 22575 after the intervention (2009). After the intervention, the GPs significantly reduced the percentage of consultations resulting in an antibiotic prescription. In patients with lower RTI the GPs in Lithuania reduced the prescribing rate by 42%, in Russia by 25%, in Spain by 25%, and in Argentina by 9%. In patients with upper RTIs, the corresponding reductions in the antibiotic prescribing rates were in Lithania 20%, in Russia 15%, in Spain 9%, and in Argentina 5%.

**Conclusion:**

A multifaceted intervention programme targeting GPs and patients and focusing on improving diagnostic procedures in patients with RTIs may lead to a marked reduction in antibiotic prescribing. The pragmatic before-after design used may suffer from some limitations and the reduction in antibiotic prescribing could be influenced by factors not related to the intervention.

## Background

Excessive and inappropriate use of antibiotics is an important reason for development of bacterial resistance [1-3]. Countries with a high use of antibiotics have a higher rate of resistance than countries with a low use [4]. As antibiotic resistance may spread across borders, high prevalence countries may serve as a source of bacterial resistance for countries with a low prevalence. Therefore, bacterial resistance is an important issue with a potentially serious impact on all countries.

Infections caused by resistant bacteria lead to increased mortality, prolonged hospital stay and increased costs [5,6]. A cornerstone of efforts to control antibiotic resistance is to improve the quality of antibiotic prescribing in primary health care, as more than 90% of antibiotics are prescribed by GPs. Approximately 70% of antibiotics prescribed in general practice are for respiratory tract infections (RTIs) [7,8]. However, the majority of RTIs (90%) are caused by virus and in these cases antibiotics are unlikely to have any clinical benefit. Studies of the management of RTIs show that a considerable number of antibiotic prescriptions are neither necessary nor appropriate [9,10]. Most RTIs are harmless and self-limiting and nearly all patients recover without any specific treatment. Antibiotic treatment may thus be superfluous, and in some cases it may be directly harmful due to adverse effects. Even if the aetiology is bacterial, antibiotics only slightly modify RTIs, particular in patients with upper RTIs [11,12].

Studies comparing bacterial resistance in various European countries have clearly documented that the prevalence of resistant strains is correlated with the consumption of antibiotics [4,13]. Until recently, the rates of antibiotic resistance in the northern European countries have remained low. However, the rates of resistance in the southern European countries are reaching alarming levels. The different antibiotic prescribing rates between countries may be due to discrepancies in national recommendations, different health care systems, different treatment traditions, different culture, different patient expectations or different impact of marketing by pharmacies and pharmaceutical companies. Several initiatives have been taken to reduce the inappropriate use of antibiotics in primary health care, but only few have achieved positive results. According to a review from the Cochrane Library, multifaceted interventions seem to be more effective than singular interventions [14]. However, only a few multifaceted interventions targeting treatment of RTIs have been performed, and we need information about the effect of multifaceted interventions in countries with different practice settings.

The aim of this study was to evaluate the impact of a multifaceted intervention programme focusing on appropriate antibiotic treatment of RTIs and targeting general practitioners and patients in general practice. The project was performed in six countries with different primary health care settings of and different prevalence of antibiotic resistance.

## Methods

Detailed information about the study method and the intervention can be found in the study protocol, published in BMC Family practice[15]. Briefly, data were obtained from GPs in two Nordic countries (Denmark and Sweden), two Baltic Countries (Lithuania and Russia) and two Hispano-American countries (Spain and Argentina). Symptoms, signs, investigations, diagnosis, assumed etiology and choice of treatment were registered for all patients with RTI during 3 weeks in the winter months of 2008 and 2009. Patients were registered using a prospective self-registry methodology based on a chart filled by the GP during the consultation [16].

Shortly after the first registration the GPs were invited to follow-up meetings where they received individual prescriber feedback and identified potential quality problems. Afterwards, they were offered an intervention programme that included the following elements:

• Training course on appropriate use of antibiotics for RTIs

• Clinical guidelines with recommendations for diagnosis and treatment of RTIs.

• Posters for waiting rooms, focusing on the appropriate use of antibiotics

• Brochures and handouts to patients about prudent use of antibiotics

• Access to Point of care (POC) tests: Strep A and C-Reactive Protein (CRP)

• Training in use and interpretation of POC tests

After the intervention, the GPs performed the second registration during a 3-week winter period one year after the first registration. For each of the involved countries we compared the antibiotic prescribing for upper and lower RTIs before and after the intervention. Furthermore, we investigated if the intervention had any influence on the choice of antibiotic.

All patient registration data were treated confidentially according to the law on protection of sensitive data and the project was conducted in accordance with the EU Directive of good clinical practice (EU Directive 2001/20/EC).

Patients registered during the study were informed about the objective of the project and they were told that specific clinical information related to the consultation was entered into a multinational database. However, no electronic patient identifier was used; patients were registered by age and sex only. The database did not contain any person-identifier or other information that could be used to identify individual registry patients. The study did not involve any randomization of patients. The protocol was submitted to a legally constituted ethics committee and deemed exempt from review (The Scientific Ethical Committee for the County of Vejle and Funen, Denmark)

Data were analyzed by the statistical program Stata, version 11. We used 95% confidence intervals (CI), adjusted for clustering to GPs.

## Results

### General practitoners

National coordinators from each of the participating countries invited local GPs to participate by e-mail, telephone or personal contact. The results presented in this paper are based on data from GPs (n = 440) participating in both registration periods (2008 and 2009). The GPs came from the following countries: Argentina (n = 48), Denmark (n = 78), Lithuania (n = 28), Russia (n = 37), Spain (210) and Sweden (39).

Table 1 shows the characteristics of the GPs participating in the study. Median age of the GPs ranged from 41 years (Argentina) to 55 years (Denmark and Sweden). In all countries, except Denmark and Sweden, the majority of GPs were women. There was a great discrepancy in the median number of years the GPs had worked in practice, ranging from 2 years (Russia) to 18 years (Spain). The majority of GPs worked in group practices comprising more than four GPs. The median number of patients listed per GP ranged from 1229 (Argentina) to 2272 (Russia). Workload, expressed by the number of consultations per day, ranged from 10 (Sweden) to 40 (Spain) and correspondingly the median number of minutes per consultation ranged from 6 minutes (Spain) to 20 minutes (Sweden).

**Table 1:** Characteristics of the GPs

Country	No of GPs	No of women (%)	Median age (iqr)	Median number of years (iqr) working in general practice	Number of GPs (%) working in single handed practice	Median number of GPs (iqr) working in group practices	Median number of patients (iqr) listed per GP	Median number of working hours per day (iqr)	Median number of minutes per cons (iqr)	Median number of cons per day (iqr)
**Argentina**	48	32 (66)	41 (34-47)	9 (4-25)	24 (53)	3 (2-4)	1229 (450-3416)	6 (4-8)	15 (10-20)	20 (15-30)

**Denmark**	78	38 (49)	55 (45-59)	14 (5-22)	30 (38)	3 (2-4)	1300 (1229-1450)	8 (8-8)	15 (10-15)	25 (22-28)

**Lithuania**	28	23 (82)	48 (43-53)	7 (6-11)	3 (11)	4 (3-6)	1480 (893-1500)	6 (5-7)	15 (13-16)	25 (20-30)

**Russia**	37	32 (86)	52 (45-55)	2 (1-4)	12 (43)	4 (4-6)	2272 (1557-2916)	7 (7-8)	15 (12-18)	24 (20-25)

**Spain**	210	127 (62)	49 (44-52	18 (12-21)	12 (6)	13 (10-17)	1694 (1500-1923)	6 (5-7)	6 (5-7)	40 (35-45)

**Sweden**	39	18 (46)	55 (47-60)	17 (9-22)	0 (0)	6 (4-8)	1708 (1467-2098)	8 (7-8)	20 (20-20)	10 (9-12)

**Total**	440	270 (62)	50 (43-54)	15 (7-20)	81 (19)	8 (4-24)	1627 (1385-1886)	7 (5-8)	10 (6-15)	30 (21-40)

### Patients

A total of 47011 patients were registered; 24436 before the intervention (2008) and 22575 after the intervention (2009) (Table 2). The majority of patients were women. The number of days with symptoms before contact to the GPs ranged from 2 days (Argentina and Russia) to 6 days (Sweden).

**Table 2:** Characteristics of patients

Country	2008				2009			
	Number of patients registered	Percentage of women (95% CI)	Median age (iqr)	Median number of days with symptoms before first consultation (iqr)	Number of patients registered	Percentage of women (95% CI)	Median age (iqr)	Median number of days with symptoms before first consultation (iqr)

**Argentina**	3499	53 (51-55)	11 (3-29)	2 (2-3)	3641	53 (52-55)	20 (9-36)	2 (1-3)

**Denmark**	2881	59 (57-60)	25 (4-46)	4 (2-7)	3706	57 (56-59)	25 (4-47)	4 (3-7)

**Lithuania**	2517	54 (52-55)	14 (6-30)	3 (2-4)	1976	54 (52-56)	13 (4-26)	3 (2-4)

**Russia**	3591	54 (52-55)	25 (12-43)	2 (1-3)	3284	53 (52-55)	26 (13-46)	2 (2-3)

**Spain**	10909	59 (58-60)	43 (30-62)	3 (2-5)	9073	59 (58-60)	43 (29-62)	3 (2-5)

**Sweden**	1039	55 (52-58)	17 (4-47)	5 (3-10)	895	56 (53-59)	20 (3-47)	6 (3-10)

**All**	24436	57 (56-57)	32 (15-52)	3 (2-5)	22575	56 (56-57)	31 (15-52)	3 (2-5)

### Prescribing rates before and after intervention

**Figure 1:**
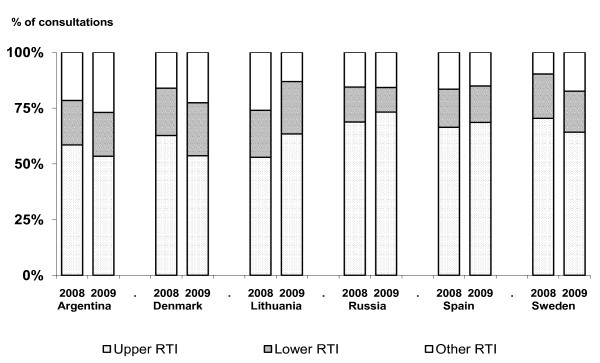
**Level of infection in patients with respiratory tract infections (RTIs) in general practice before and after intervention**.

**Figure 2:**
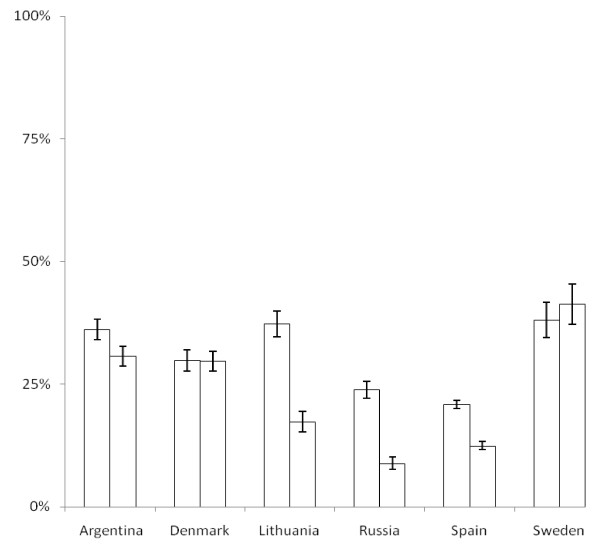
**Prescription rate of antibiotics (% of consultations resulting in an antibiotic prescription) in patients with upper respiratory tract infections before and after the intervention**.

**Figure 3:**
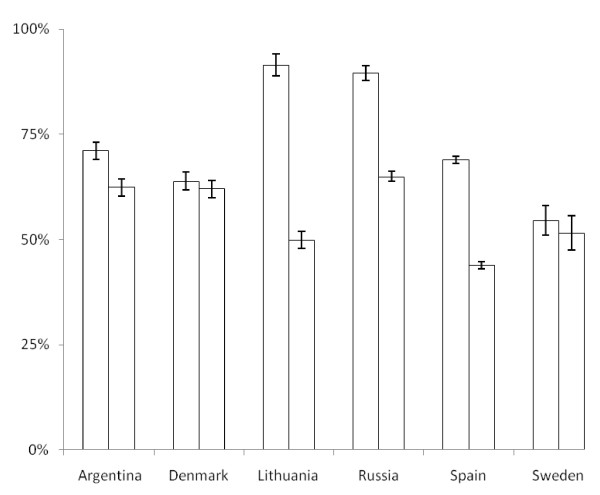
**Prescription rate of antibiotics (% of consultations resulting in an antibiotic prescription) in patients with lower respiratory tract infections before and after the intervention**.

Figure 1 shows the distribution of upper and lower RTIs before and after the intervention. In all countries, patients with upper RTIs represented the majority of consultations. The rate of antibiotic prescribing was highest for lower RTIs, but there were big differences in prescribing rates between GPs from different countries (Figures 2 and 3). Before the intervention, the highest prescribing rates for lower RTIs were found in Lithuania, where about nine out of ten consultations resulted in an antibiotic prescription.

A significant reduction in the antibiotic prescribing rate was found in the Baltic countries and Hispano-America, while no significant change was seen in the Nordic countries. For patients with lower RTIs, GPs from Lithuania reduced their prescribing rate by 42% (CI: 36%-47%), GPs from Russia by 25% (CI: 19%-30%), GPs from Spain by 25% (CI: 22%-28%), and GPs from Argentina by 9% (CI: 4%-14%). For patients with upper RTIs, the antibiotic prescribing in Lithania was reduced by 20% (CI: 17%-23%), in Russia by 15% (CI: 13%-17%), in Spain by 9% (CI: 7%-10%) and in Argentina by 5% (CI: 3%-8%).

There were huge discrepancies between countries in the choice of antibiotics for RTIs (Tables 3 and 4). In patients with upper RTIs, GPs in the Nordic countries preferred penicillin-V while GPs in Argentina, Lithuania, Russia and Spain preferred amoxicillin. However, after the intervention a considerable increase in the prescribing rate of penicillin-V for all patients with upper RTIs was registered. In patients with lower RTIs, GPs in the Nordic countries preferred penicillin-V while GPs in Argentina, Lithuania, Russia and Spain preferred amoxicillin or amoxicillin with clavulanic acid. The prescribing rate of tetracycline, quinolone, and cephalosporin was generally low. However, in Sweden a considerable part of patients with RTIs were treated with tetracycline, in Spain with quinolone, and in Russia with cephalosporin. In Denmark, many RTIs were treated with macrolide, but a marked reduction was found after the intervention.

**Table 3:** Choice of antibiotic (%) in patients with upper respiratory tract infections before and after the intervention

	Before intervention (95% CI)*							
	Penicillin-V	Amoxicillin	Amoxicillin-Clavulanic acid	Macrolide	Quinolone	Tetracycline	Cephalosporin	Other

**Argentina**	15.3 (8.2-22.4)	54.7 (38.3-71.1)	16.9 (5.0-28.8)	2.3 (0.7-4.5)	1.5 (0-3.0)	0.1 (0-0.4)	4.1 (0-8.5)	10.0 (0-21.1)

**Denmark**	79.6 (74.0-85.3)	9.2 (5.9-12.6)	0.2 (0-0.5)	7.2 (4.7-9.8)	0.2 (0-0.5)	0.2 (0-0.5)	0 (0-0)	3.9 (0.3-7.5)

**Lithuania**	24.2 (16.7-31.8)	37.6 (26.0-49.1)	18.1 (13.3-22.8)	6.5 (4.2-8.9)	0 (0-0)	1.8 (0-3.8)	10.9 (4.5-17.4)	0.8 (0-1.6)

**Russia**	9.0 (3.4-14.6)	42.5 (31.3-53.8)	29.2 (18.6-39.8)	14.1 (5.8-22.3)	1.9 (0-4.0)	1.4 (0-4.0)	1.8 (0.6-2.9)	0.5 (0-1.0)

**Spain**	10.1 (7.1-13.2)	34.8 (30.2-39.4)	32.2 (27.6-36.7)	9.0 (6.2-11.9)	1.8 (0.9-2.8)	0.1 (0-0.2)	3.8 (2.6-5.1)	8.8 (3.3-14.4)

**Sweden**	78.1 (70.6-85.5)	6.8 (3.5-10.2)	0 (0-0)	2.9 (0.7-5.1)	0 (0-0)	6.8 (0.3-11.0)	2.1 (0.1-4.2)	3.2 (0.9-5.6)

	After intervention (95% CI)*							

**Argentina**	31.2 (22.6-39.8)	40.1 (26.6-53.6)	8.1 (3.5-12.7)	2.0 (0-4.2)	0.5 (0-1.2)	0.3 (0-1.0)	0.5 (0-1.2)	22.4 (7.2-37.7)

**Denmark**	83.7 (79.4-87.9)	10.0 (6.2-13.9)	2.0 (0.2-3.9)	3.0 (1.7-4.5)	0.3 (0-0.8)	0 (0-0)	0 (0-0)	1.2 (0.1-2.2)

**Lithuania**	68.5 (58.5-78.6)	17.6 (8.4-26.8)	2.8 (1.2-4.3)	7.9 (4.0-11.7)	0 (0-0)	0.5 (0-1.4)	0.5 (0-1.4)	2.3 (0-6.1)

**Russia**	19.4 (8.8-30.1)	55.0 (43.5-66.4)	12.8 (5.2-20.4)	5.7 (0-12.5)	0 (0-0)	0.9 (0-2.1)	4.3 (0.7-7.8)	1.9 (0-4.5)

**Spain**	31.0 (26.2-35.9)	29.8 (25.0-34.5)	26.4 (22.0-30.7)	4.7 (2.9-6.4)	1.7 (0.7-2.6)	0 (0-0)	2.7 (1.3-4.1)	4.1 (1.1-7.2)

**Sweden**	75.9 (70.2-81.7)	7.1 (3.7-10.7)	1.2 (0-2.7)	3.8 (1.3-6.3)	0 (0-0)	6.7 (3.4-10.1)	2.5 (0.5-4.6)	2.9 (1.1-4.8)

*: Adjusted for clustering to GPs

**Table 4:** Choice of antibiotic (%) in patients with lower respiratory tract infections before and after the intervention

	Before intervention (95% CI)*							
	Penicillin-V	Amoxicillin	Amoxicillin-Clavulanic acid	Macrolide	Quinolone	Tetracycline	Cephalosporin	Other

**Argentina**	4.2 (1.6-6.9)	52.6 (38.6-66.7)	24.5 (13.8-35.2)	9.7 (5.5-14.0)	1.6 (0-3.5)	0 (0-0)	4.6 (1.1-8.2)	10.3 (1.3-19.3)

**Denmark**	60.0 (55.3-65.7)	13.6 (10.0-17.2)	1.3 (0-2.9)	23.1 (17.6-28.5)	0.8 (0-1.8)	1.0 (0-2.4)	0 (0-0)	0.8 (0-1.6)

**Lithuania**	6.9 (1.7-12.2)	33.4 (21.9-44.9)	25.4 (17.3-33.5)	19.7 (14.8-24.7)	1.7 (0.3-3.1)	1.8 (0.3-3.5)	10.7 (3.6-17.9)	1.3 (0-2.6)

**Russia**	4.7 (0.4-9.1)	33.7 (24.1-43.4)	28.0 (17.1-38.9)	13.8 (5.8-21.7)	4.7 (0-9.5)	0.4 (0-1.0)	20.2 (13.1-27.2)	0.8 (0-1.7)

**Spain**	0.2 (0-0.5)	19.3 (15.8-22.8)	44.9 (40.7-49.1)	13.5 (10.6-16.4)	16.0 (12.9-19.1)	0.4 (0-0.9)	3.6 (2.3-5.0)	2.5 (0.6-4.3)

**Sweden**	30.1 (19.2-40.9)	14.2 (8.0-20.3)	0 (0-0)	6.2 (1.9-10.4)	0 (0-0)	48.7 (39.1-58.3)	1.8 (0-4.1)	0 (0-0)

	After intervention (95% CI)*							

**Argentina**	3.3 (0.5-6.2)	35.5 (26.3-44.6)	34.6 (25.4-43.8)	19.4 (9.8-29.0)	2.4 (0-5.3)	0.9 (0-1.8)	4.9 (0.4-9.4)	21.4 (2.0-40.9)

**Denmark**	70.7 (65.7-75.8)	10.8 (6.7-14.9)	6.2 (3.4-9.0)	11.1 (8.2-14.1)	0 (0-0)	0.2 (0-0.5)	0 (0-0)	0.9 (0-1.8)

**Lithuania**	38.1 (25.1-51.0)	22.9 (13.8-32.1)	6.1 (0.2-12.0)	22.1 (14.3-29.9)	0 (0-0)	3.0 (0.9-5.2)	5.2 (2.0-8.4)	3.4 (0-7.7)

**Russia**	10.7 (1.8-19.4)	47.9 (35.0-60.7)	17.9 (8.1-27.8)	6.4 (1.9-10.9)	2.5 (0-5.2)	0 (0-0)	15.8 (6.7-24.9)	0.4 (0-1.3)

**Spain**	0.5 (0-1.0)	22.5 (18.0-27.0)	42.8 (37.7-48.0)	12.9 (9.7-16.0)	16.1 (12.7-19.4)	0 (0-0)	3.9 (2.0-5.9)	1.8 (0.9-2.8)

**Sweden**	43.5 (29.8-57.3)	10.6 (3.3-17.9)	0 (0-0)	5.9 (1.5-10.2)	0 (0-0)	37.6 (23.5-51.8)	0 (0-0)	2.3 (0-5.6)

*: Adjusted for clustering to GPs

## Discussion

This study showed that a combined intervention programme targeting GPs and patients and focusing on improving diagnostic procedure and treatment in patients with RTIs led to a marked reduction in antibiotic prescribing and a significant change in the choice of antibiotics. A considerable reduction in antibiotic prescribing was found in Argentina, Lithuania, Russia and Spain. In Denmark and Sweden we found no significant changes in the overall use of antibiotics, but marked changes were found related to the choice of antibiotics.

The intervention aimed to help GPs to distinguish between viral and bacterial aetiology. All practices were offered access to POC tests (StrepA and CRP) and the practice staff was instructed how to interpret the results. GPs were encouraged to employ a rational use of antibiotics according to the HAPPY AUDIT guidelines[15], and they were requested only to prescribe antibiotics to patients with a suspected bacterial aetiology.

All GPs were exposed to the multifaceted intervention activities, and based on the results in this study it is not possible to identify the elements that had the highest impact on the prescibing pattern. The majority of GPs (GPs from Argentina, Spain, Russia amd Lithuania) did not have access to POC test before the intervention, while most GPs from the Nordic countries used POC tests routinely. The marked effect of the intervention found outside the Nordic countries may to a certain extent be due to the introduction of POC tests in practice.

Our data must, however, be interpreted with caution due to a number of limitations. GPs participated on a voluntary basis and probably their prescribing habits may not represent the average use of antibiotics in their country [17]. GPs that were willing to register their antibiotic prescribing may have been more interested in quality development and research than GPs in general. Furthermore, they were willing to dedicate sufficient time to complete patient reports without economic incentives. The amount of time involved in this project could be considered to be a prominent barrier to participation, as GPs might find it difficult to dedicate the time in their daily work. However, earlier studies using the same type of data registration did not find it very time-consuming. Each registration takes less than 2 minutes, but the GPs also needed to allocate sufficient time for the subsequent courses and other activities planned during the intervention period.

Another limitation which should be taken into account is the fact that performing a registration on antibiotic use may in itself influence the prescribing habits. However, studies have shown that the reliability of this methodology applied in different countries is high and findings are correlated with the real prescribing in practice [16].

In our study, we asked the GPs to register what happened during the consultation, but patients were not followed after the consultation and thus we have no knowledge about the consequence of reducing antibiotic prescribing for the patients involved. From a theoretical point of view, the decision to treat should be taken after a diagnosis has been established. In general practice, however, the diagnostic procedures and the decision to treat are intricately linked. The GP may decide whether or not to prescribe an antibiotic at the same time, or even before, he classifies a specific diagnosis to the patient. After making the decision to prescribe the GP may thus adjust the diagnosis to fit the decision about treatment. This may lead to a diagnostic misclassification bias. However, this potential bias will affect the validity of the diagnosis both before and after the intervention and it only has a small likelihood of influencing the effect of the intervention.

Due to the limited time allocated for the registration process in practice only the typical signs and symptoms of RTIs according to the medical literature were recorded. This may lead to some limitations. The before-after design without a control group suffers from some limitations due to the fact that changes in antibiotic prescribing could be due to factors other than the intervention performed by the investigators. Non-biomedical factors that might represent powerful predictors of antibiotic prescription such as market regulation and socio-economic factors were not taken into account in this study.

This is a pragmatic study where registration of patients was performed in a natural practice setting. Patients were not informed about the project prior to the consultations. GPs participating in the audit were not allocated extra time for consultations, and they were not able to make considerable changes in their practice activities during the 3 weeks of registration. Thus, they attended the same patients as if they were not participating in the study. Therefore, it is most likely that our results can be extrapolated to other areas and practices with similar settings.

## Conclusion

We found that a combined intervention programme targeting GPs and patients and focusing on improving diagnostic procedures in patients with RTIs led to a marked reduction in antibiotic prescribing. The pragmatic before-after design without control group suffers from some limitations due to the fact that changes in antibiotic prescribing could be influenced by factors not related to the intervention. All GPs were exposed to the multifaceted intervtention activities, and it is not possible to identify which of the elements that had highest impact on the prescribing pattern.

## Competing interests

The authors declare that they have no competing interests.

## Authors' contributions

LB, CL and BGL-V drafted the manuscript. All the authors participated in data production and offered critical revisions of the manuscript.

## Pre-publication history

The pre-publication history for this paper can be accessed here:

http://www.biomedcentral.com/1471-2296/12/52/prepub
